# Diagnosing and managing prescription opioid use disorder in patients prescribed opioids for chronic pain in Australian general practice settings: a qualitative study using the theory of Planned Behaviour

**DOI:** 10.1186/s12875-024-02474-6

**Published:** 2024-07-03

**Authors:** HHK Wilson, B. Harris Roxas, N. Lintzeris, MF Harris

**Affiliations:** 1 Drug and Alcohol Services, South East Sydney Local Health District, Sydney, NSW Australia; 2 School of Population Health, University of New South Wales, Sydney, NSW Australia; 3 Department Addiction Medicine, University of Sydney, Sydney, NSW Australia; 4 NSW Drug and Alcohol Clinical Research and Improvement Network (DACRIN), NSW Health, Sydney, NSW Australia; 5 Centre for Primary Health Care and Equity (CPHCE), University of New South Wales, Sydney, NSW Australia

**Keywords:** General practice, Primary care, Chronic (disease)/pain, Substance use disorder, Opioid use disorder, Opioid dependence, Diagnosis/differential, Prescription/drugs, Qualitative, Theory of planned behaviour.

## Abstract

**Background:**

Chronic pain is a debilitating and common health issue. General Practitioners (GPs) often prescribe opioids to treat chronic pain, despite limited evidence of benefit and increasing evidence of harms, including prescription Opioid Use Disorder (pOUD). Australian GPs are worried about the harms of long-term opioids, but few are involved in the treatment of pOUD. There is little research on GPs’ experiences diagnosing and managing pOUD in their chronic pain patients.

**Methods:**

This qualitative research used semi-structured interviews and a case study to investigate GPs’ experiences through the lens of the Theory of Planned Behaviour (TPB). TPB describes three factors, an individual’s perceived beliefs/attitudes, perceived social norms and perceived behavioural controls. Participants were interviewed via an online video conferencing platform. Interviews were transcribed verbatim and thematically analysed.

**Results:**

Twenty-four GPs took part. Participants were aware of the complex presentations for chronic pain patients and concerned about long-term opioid use. Their approach was holistic, but they had limited understanding of pOUD diagnosis and suggested that pOUD had only one treatment: Opioid Agonist Treatment (OAT). Participants felt uncomfortable prescribing opioids and were fearful of difficult, conflictual conversations with patients about the possibility of pOUD. This led to avoidance and negative attitudes towards diagnosing pOUD. There were few positive social norms, few colleagues diagnosed or managed pOUD. Participants reported that their colleagues only offered positive support as this would allow them to avoid managing pOUD themselves, while patients and other staff were often unsupportive. Negative behavioural controls were common with low levels of knowledge, skill, professional supports, inadequate time and remuneration described by many participants. They felt OAT was not core general practice and required specialist management. This dichotomous approach was reflected in their views that the health system only supported treatment for chronic pain or pOUD, not both conditions.

**Conclusions:**

Negative beliefs, negative social norms and negative behavioural controls decreased individual behavioural intention for this group of GPs. Diagnosing and managing pOUD in chronic pain patients prescribed opioids was perceived as difficult and unsupported. Interventions to change behaviour must address negative perceptions in order to lead to more positive intentions to engage in the management of pOUD.

**Supplementary Information:**

The online version contains supplementary material available at 10.1186/s12875-024-02474-6.

*‘Fear is the cheapest room in the house. I’d like to see you in better living conditions’* Hafiz, Persian mystic and poet.

## Background

A leading cause of disability worldwide [1], chronic pain is defined as persistent pain continuing for longer than 3–6 months and occurring on most days [2]. It is a complex condition, *‘an individual, multifactorial experience influenced by culture, previous pain events, beliefs, expectations, mood and resilience’* [3]. It has been estimated that 20% of Australians over age 45 experience chronic pain [2]. Nearly one fifth of patients seen by their general practitioner (GP) are suffering chronic pain [4]. Rates of opioid prescribing by Australian GPs for chronic pain are high [2]. One or more opioid prescriptions, mostly oxycodone, were provided to 3.1 million (13%) of the Australian population in 2016-17 with 1.5% (46,500 people) taking them on a daily basis [5].

Long term opioid use, that is, daily use on most days for more than 3 months [6, 7], is associated with increasing evidence of significant harms and limited effectiveness for chronic pain [8–11]. Risky opioid use or non-medical use of opioids in people prescribed opioids is common [12]. Opioid risk increases with dose and length of use [13, 14]. Each day, in Australia, three people die and 150 are hospitalised due to pharmaceutical opioid overdose [5]. Other significant health risks include hyperalgesia (increased pain sensitivity), endocrine abnormalities, falls, fractures, motor vehicle accidents, aberrant medication behaviours and medication on-selling or sharing [8, 15–17]. Nearly one in 10 people prescribed opioids for chronic pain in Australia meet criteria for Opioid Use Disorder (OUD) [14]. OUD is categorised by the American Psychiatric Association in the Diagnostic and Statistical Manual (DSM-5-TR) as a pattern of opioid use with clinically significant impacts [18]. Opioid Agonist Treatment (OAT), is an evidence-based treatment for OUD and prescription OUD (pOUD) and includes two opioid medications, methadone and buprenorphine [19, 20]. In Australia, state based OAT programs allow GPs to diagnose and prescribe methadone and buprenorphine for OUD [21]. This treatment, like many other chronic conditions, can be appropriately managed for many patients in general practice [22].

In the UK, 50% of GPs prescribed OAT in 2005 [23]. While in Ireland, 54% of GPs trained in the management of OUD in 2018 [24, 25]. In 2022, in contrast, 2,352 private prescribers (mostly GPs) provided OAT Australia wide [26]. With 31,926 GPs working in Australia in 2022 [27], this suggests low engagement, with only 7% of Australian GPs providing OAT. This is supported by research that suggests Australian GP assessment of pOUD, management with OAT and referrals for OUD to specialist Alcohol and Other Drugs services are low [28–30]. Australian and international literature suggests that issues of stigma, poor remuneration, low knowledge, confidence, and lack of specialist support adversely affect GP involvement in OAT [31–34]. Our recent scoping review found that current published literature described GPs’ concern regarding risk of prescription opioid overdose, addiction and diversion, but screening for pOUD was haphazard [35]. We could find no literature that explored Australian GPs’ experience of diagnosing and managing pOUD in their chronic pain patients for whom they prescribed opioids [35].

### Research aim

This research aims to gain an in-depth understanding of GPs’ attitudes and experience diagnosing and managing pOUD in their patients’ prescribed opioids for chronic pain in the community general practice setting in the state of New South Wales, Australia.

## Methods

### Study design and setting

This qualitative study used semi-structured interviews to explore GPs’ experience of diagnosing and managing pOUD in patients prescribed opioids for chronic pain. The semi-structured interview method was chosen as it is useful to investigate individuals’ subjective experience [36]. We used the Theory of Planned Behaviour (TPB) to frame, code and investigate the issues [37].

**Fig. 1:**
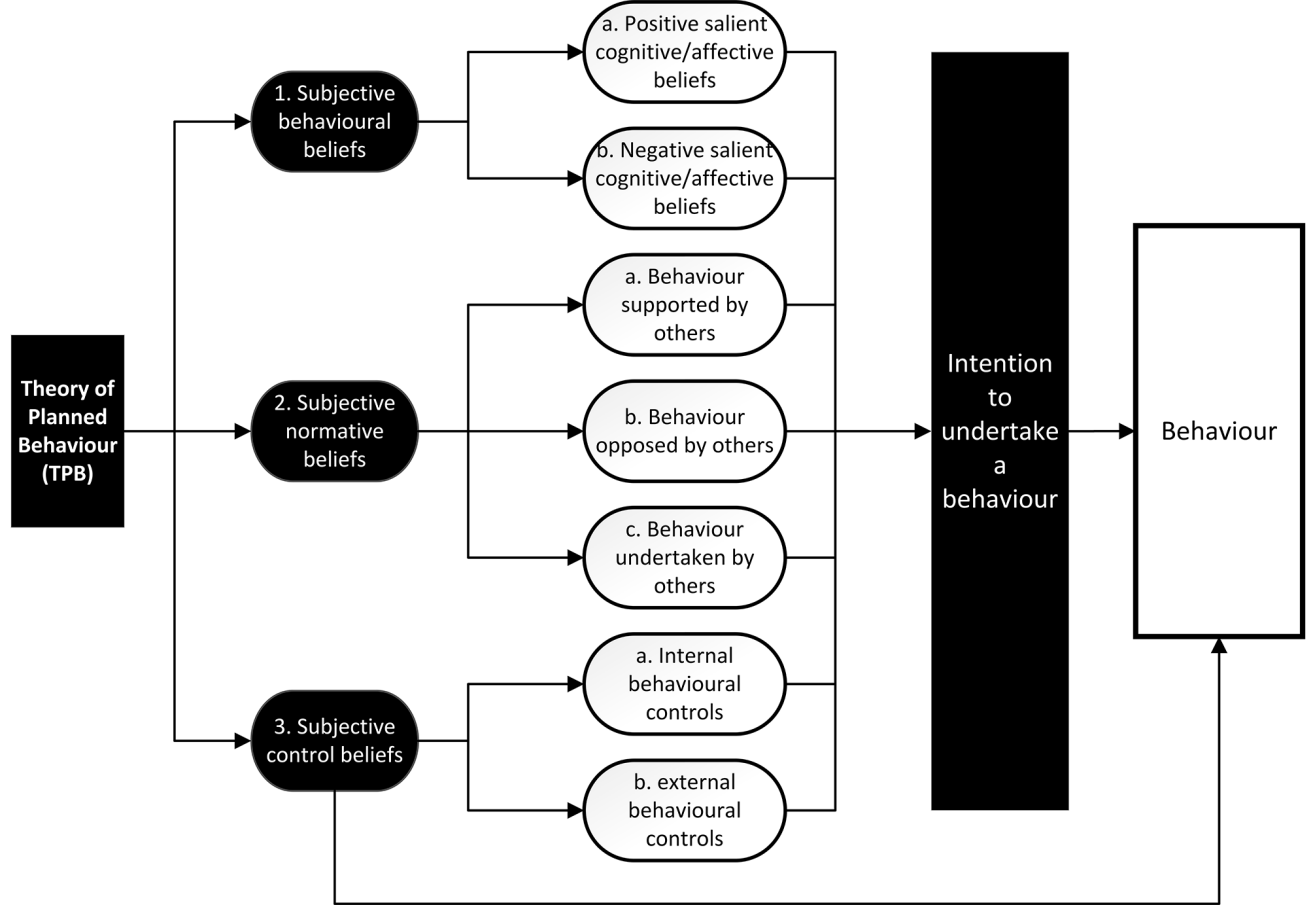
TPB factors and how they affect behaviour

TPB assesses an individual’s perception of the issues that surround a decision to undertake a behaviour and elucidates the factors that increase or decrease intention to undertake this behaviour. It describes three subjective factors perceived by individuals: subjective behavioural, normative and control beliefs. Behavioural beliefs are guided by emotions (affect) and thoughts (cognition) and inform positive and negative attitudes. Normative beliefs are guided by perceived social norms; what a person thinks others do themselves and whether others support or oppose the individual undertaking the behaviour. Control beliefs describe barriers or facilitators, both internal, e.g., knowledge, and external e.g., time. The negative or positive strengths of these three factors affect intention to undertake a behaviour, which influences whether the actual behaviour is performed [37, 38]. The theory also suggests that subjective control beliefs can directly influence behaviour [39]. See Fig. 1.

It is important to note that TPB only addresses individuals’ perceptions. It is not a model for behaviour change nor does it systematically address how systems affect behaviour.

The interview guide asked about participant’s experience with chronic pain and pOUD through the lens of TPB and their awareness of any policies or strategies to support GP opioid prescribing. (See supplemental File 1 – interview guide).

A two-part case study supported the interview guide. Part one, depicted a 42-year-old woman prescribed opioids in hospital after acute pelvic and spinal injury some years previously, who attends her GP practice regularly for opioid prescriptions. Part two describes signs and symptoms that suggest pOUD. (See Table 1).

**Table 1:** Case Study

**Part 1** Judy is a 48-year-old woman. She has been seen in your practice over several years. She has 2 children, aged 12 and 15 and lives with her husband. She has a history of post-natal depression, endometriosis, and psoriasis. Four years ago, she was involved in a motor vehicle accident and fractured her pelvis. Since this time, she has suffered daily severe back and pelvic pain. She was commenced on oxycodone in hospital and discharged on 80 mg BD. She has continued this since that time, attending your practice for regular prescriptions.
**Part 2** You are concerned about the dose Judy is taking and look back over her past records. You note that she lost a prescription about 6 months ago and had this reissued. She attends every 3 weeks for prescriptions. She has recently complained that her pain is really troubling her and requested an increase in dose. You review her prescriptions and find that her daily dose has escalated from 160 mg to 240 mg a day. In consultation Judy expresses her concerns about the medication. She would like to stop it. She finds she feels ‘fuzzy ‘and her family complain that she seems disconnected from them. Her husband is worried about her. She has not returned to work and her pain is still bad. She has tried to cut down but feels nauseous, gets diarrhoea and stomach cramps, doesn’t sleep, and says ‘I’m watching the clock’ for the next dose.

### Participant recruitment

GPs in New South Wales (NSW), Australia, were recruited via Primary Healthcare Networks, an Australian GP Facebook page called ‘GPs Down Under’ and via snowballing by email. All interviews were undertaken via a video conferencing platform (Zoom) from May to September 2021 by the lead author (HW). To be eligible, participants needed to be federally registered as medical practitioners, and working in the community primary care setting in NSW. The study was limited to NSW due to variations in opioid and OAT prescribing legislation and accreditation in each state/territory in Australia.

### Data collection

The interviews were audio recorded, transcribed verbatim and de-identified. Data were stored on a secure server. A reflective journal helped support the audit trail.

### Data analysis

The data were analysed deductively using the mid-level theoretical framework of TPB [37] and inductively with open coding, including thematic analysis [40]. Top-level codes were grouped under the ‘a priori’ conceptual categories of subjective behavioural beliefs, subjective normative beliefs and subjective control beliefs while open coding allowed the analysis of other aspects that were seen to be important [41]. Higher-order concepts were interpreted through testing of codes, reiterative reflection, and extensive rereading of transcripts. The data was managed via QSR N-Vivo software. Authors HW and BHR reviewed the transcripts to support data accuracy and integrity.

COREQ checklist for qualitative reporting [42] are included in supplemental file 2.

### Researcher positionality

We used an interpretive description approach. This emphasised analysis of in-depth contextual description, drawing on interpretation, clinical and research experience in order to understand practice-based issues [43, 44]. The lead researcher (HW) is a GP, addiction specialist and PhD student with extensive clinical experience managing chronic pain, prescribing opioids and OAT. The senior researchers include a GP (MH), a primary care researcher (BHR) and an addiction specialist (NL).

### Ethics approval

Ethics approval was obtained from the Human Research Ethics Committee of the South Eastern Sydney Local Health District (HREC 18/018 (LNR/18/POWH/156) and University of NSW HREC18/018. All methods were carried out in accordance with relevant guidelines and regulations and informed consent was obtained from all participants involved in this study.

## Results

Twenty-four GPs took part in the study. They all saw patients with chronic pain and 23 reported currently prescribing opioids for this indication. Fifteen were female. There was a wide range of ages and practice experience. Participants worked across metropolitan, regional and rural NSW [45]. Five prescribed OAT currently (one was a GP and a Fellow of the Chapter of Addiction Medicine) and 2 GPs reported prescribing OAT in the past but not currently. (See Table 2).

**Table 2:** Participant demographics

**Participants**	(*n* = 24)
**Sex**	Female = 15 (63%) Male = 9 (38%)
**Age**	25–34 = 7 (29%) 35–44 = 11 (46%) 45–54 = 4 (17%) 55 + = 2 (8%)
**Culturally and Linguistically Diverse (CALD) background**	Yes = 8 (34%) No = 16 (66%)
**Length of GP experience**	GP Registrar = 1 (4%) New Fellow* = 9 (38%) Established GP = 14 (58%)
**Length in current practice**	< 3 yrs. = 7 (29%) 3–10 yrs. = 13 (54%) 11–20 yrs. = 2 (8%) 21–30 yrs. = 0 > 30 yrs. = 2 (8%)
Postgraduate training in pain (masters, hospital placement or CPD**)	Yes = 13 (54%)
Postgraduate training in addiction (accredited prescribing of OAT, CPD** or Fellowship in Addiction)	Yes = 3 (13%)
**Prescribing OAT**	Ever prescribed OAT = 7 (29%) Prescribing OAT now = 5 (21%)
**Rurality**	Metro (Sydney) = 14 (58%) Regional centre = 4 (17%) Rural = 6 (25%)

*New Fellow – within 5 years of graduation from GP training

**CPD – Continuing Professional Development

This study used the three factors in TPB (see Fig. 1) to analyse the interviews, however there was an overarching universal theme of holistic and complex care.

### Holistic complex care in the general practice setting

Participants gave extensive responses to the case study patient’s presentation and her social, vocational, family, mental and physical co-morbidities. This universal approach may be linked to each participant’s identity as a GP and appeared integral to their professional approach to patients.

* ‘….how does the pain limit what she can do? how’s it affecting her relationships? What else is going on for her husband and her teenage kids?   Endometriosis* (a disorder of abnormal spread of the womb lining)*… the psoriasis* (a chronic skin condition) *… mental health issues… she’s probably perimenopausal* (the period of time around menopause)*… she hasn’t even managed to get back to work…’* (GP18, female, metro, established GP).

Participants were aware of the complexities of managing chronic pain and suggested that chronic pain rarely presented alone, and this was difficult to adequately address.

*‘No one ever comes in just for their chronic pain. And it’s a 15 minute consultation, usually that they’ve booked. And there’s a lot of other things going on….a lot of them are either too disorganized, too much going on with their life socially or within other medical conditions…’* (GP4, female, regional, new fellow).

Sitting underneath the theme of ‘holistic complex care in the general practice setting’ were the three factors of TPB.

### Subjective behavioural beliefs diagnosing and managing pOUD

Many participants sighed or paused for long periods when answering questions related to diagnosis and management of pOUD in chronic pain patients prescribed opioids.

#### Positive salient affective/cognitive (thoughts/feelings) beliefs

Some participants described positive thoughts and feelings about diagnosing and managing pOUD. This included being a good doctor, doing the right thing, achieving something difficult and appropriate treatment leading to better patient outcomes.

*‘because when you have the right diagnosis…. you have the right treatment…’* (GP1, male, rural, new fellow).

*‘…it would benefit several of my patients in real life, and it would certainly benefit Judy’* (case study patient). (GP3, female, rural, registrar)

#### Negative salient affective/cognitive (thoughts/feelings) beliefs

Drawing on past experience, most participants expressed high levels of negative thoughts and feelings when considering pOUD in chronic pain patients. They described the case study as *‘really difficult’* and a *‘heart sink patient’*, like patients they had seen in the past. Patients, whom, they had found to be time-consuming and someone they didn’t want to see or knew they would continue to think and worry about after the consultation.

*‘a demanding patient… one of those patients, … oh, I have to see her today or you’d go home, and think, oh, why did I say that, or do that. So it’s one of those patients, that you kind of dwell on before and after the consult…’* (GP10, male, rural, established GP).

Most participants described the difficulty and futility of trying to talk to chronic pain patients about changing their opioid treatment.

*‘You raised it a hundred times previously and like a broken record, you raise it again and at some point, you think, what’s the point? Like, I raised it a hundred times and it gets nowhere so why should I bother?’* (GP9, male, regional, established GP).

Many expressed a sense of nihilism, that there was not much they could do beyond prescribing opioids.

*‘…you feel like there’s nothing I can do, apart from giving them this medication…’* (GP9, male, regional, established GP).

Participants were worried that diagnosing and managing pOUD would fracture the GP-patient therapeutic alliance.

*‘… feeling like the rapport is broken, that they won’t come back and see you and you have no idea what happens to them …’* (GP16, female, metro, new fellow).

Some participants expressed regret prescribing opioids and described feeling guilty and complicit. They felt a personal responsibility for opioid harms experienced by patients.

*‘…you have to come to terms with the fact that you have done something, which actually is not good health care. You know that’s a pretty sobering thing to realize that you’ve actually been complicit…’* (GP14, female, regional, established GP).

*‘I feel quite guilty when people come in and they’re like this, because we’ve started (opioids)…. and now this person is in a whole heap of trouble, and mess.*’ (GP9, female, metro, established GP).

The risks of prescription opioid overdose and withdrawal were recognised by all participants. This led to feeling overwhelmed by the situation for some participants.

*‘…if you do give them the medication you’re worried about them overdosing, if you don’t give them the medication you worry about them getting withdrawal symptoms…’* (GP17, male, metro, established GP).

Many participants described the onerous responsibility of managing pOUD long term if they diagnosed it, as they believed management was going to be difficult.

*‘I don’t want to be the one to do it, because I don’t want to be the one that’s taking responsibility, I know this sounds horrible, but I really don’t want to be the one that’s taking responsibility for the ongoing care with this because I know that it’s gonna be really difficult…’* (GP20, female, new fellow, metro).

Some suggested that with all the competing demands placed on GPs, addressing pOUD was low on their priorities. They suggested that this was a group of people who appeared stable and didn’t complain about their medication. As a result, some participants suggested they found it easier to continue prescribing opioids for the management of chronic pain. The participants found considering the issue of pOUD immediately made the happy patient unhappy and took time, was complex and impossible to manage.

*‘…these people generally are stable, they’re often not complaining too much, they just pitch up every four weeks, and we, we forget actually, it becomes very low on that list of priorities, if I’m honest, I think it just sort of gets sucked up in doing everything every day, and you have to actually make that conscious decision, are you going to address this problem?’* (GP4, female, metro, established GP).

Many participants expressed a guilty relief when patients with complex chronic pain presentations stopped seeing them. They expressed concern about the risk of burnout.

*‘….you never want to be sacked by a patient, but I wasn’t disappointed….she was quite a demanding patient saturating my energy and my time…’* (GP11, male, rural, established GP).

*‘…they’re long hard consults… you risk burning out really…I don’t want to burn out by loading up my days with dealing with this…’* (GP10, male, rural, established GP).

Most participants described feeling uncomfortable and avoiding difficult conversations about pOUD with chronic pain patients. As health professionals they wanted to help and found it difficult to frame the conversation in a way that would assist the patient to reconsider their treatment.

*‘…how do I really explain that well to the patient, because a lot of them will just think, you’re not helping me, or you’re taking away something that I need. And I think that’s the hardest thing as a GP….is that you want to help. And so, if you’ve got someone saying well this is helping me and you’re taking it away, how you explain, frame that for them, I still find really difficult…’* (GP4, female, regional, established GP).

The difficulty of the conversation seemed to lead to therapeutic inertia for the participants.

*‘…and especially if I’m running late, or busy or if I’m tired, there’s a temptation to just, you know, tie them over. Yeah, not have that difficult conversation.’* (GP9, male, outer metro, established GP).

The participants with training in the management of OUD expressed similar negative experiences and attitudes.

*‘I find these patients really, really difficult. With what I feel is a reasonable amount of experience and knowledge about how to treat…I still feel uncomfortable…’* (GP3, male, metro, established GP).

Most participants noted that while the patient in the case study seemed to have some insight into their situation, this was uncommon. In their experience, patients had little insight or desire to change their medication and could not perceive doing anything differently. The discussion felt like a battle where the GP tries to discuss changing treatment and the patient defends their position.

*‘…it’s ‘why are you even asking me this question, it’s not a problem, …it’s never been a problem before?’ … they know that they have to put up a fight to get the script, because there’s a general sort of culture of ‘no I don’t want to give this medication to you’ every time. You know, every time I ask, I have to fight for it.’* (GP2, female, rural, established GP).

Prescribing opioids for chronic pain was seen as part of a GP’s role but many did not consider managing pOUD as ‘usual business’.

*‘…prescribing opiates, even large doses of opiates…the vibe is it’s a normal part of general practice, while the vibe is, I think, perhaps treating substance use disorders, and maybe particularly with opiate use disorders is not….’* (GP15, male, metro, established GP).

Some participants described the need to actively work to change their mindset, to stop and consider that the treatment they were providing could be causing harm.

*‘I remember having to stop and just go, hang on, I am giving this medication that is causing her more harm, and it was such a different mindset for me to have to just go, this is not working and it was a medication I was prescribing for her.’* (GP13, female, metro, established GP).

### Subjective normative beliefs diagnosing and managing pOUD

#### Diagnosing/managing pOUD supported by others

Many participants perceived that specialist pain and addiction services were happy for them to diagnose and manage pOUD as this would relieve pressure on their services. One participant suggested that some of their GP colleagues were supportive, but only because this meant they would not have to do this themselves. This was seen as a perverse disincentive to diagnose and manage pOUD.

*‘…it would be; ‘I’m* (The GP colleague) *really glad that you’re* (the participant) *doing this so I don’t have to do it, and then everyone would refer…rather than taking it on themselves…’* (GP2, female, rural, established GP).

#### Diagnosing/managing pOUD NOT supported by others

Some participants suggested that while they might be happy to undertake diagnosing and managing pOUD, they had to consider their colleagues who may be concerned about risks and how this would affect practice amenity and other patients’ safety. Some participants suggested that staff would not approve of people with pOUD and did not want *‘these patients’* in the practice.

*‘Changing the stigma of my* (senior) *colleague…it’s not going to be easy to change his mind about things, change his views, his perception, and he would feel like, ‘what are you turning this clinic into?’’* (GP6, male, metro, new fellow).

Most participants perceived that patients themselves did not want a pOUD diagnosis, they did not want their management to change or become part of a stigmatised patient group, they did not want referral to drug and alcohol services and did not see themselves as possibly needing a change in treatment plan, such as deprescribing or OAT.

*‘…this poor girl literally sat in my room crying, being like, “I don’t want to be labelled a druggie”….’* (GP19, female, metro, new fellow).

*‘…they don’t see themselves as someone who should be on methadone or suboxone. And there’s a lot of shame and stigma around that …’*(GP2, female, rural, established GP).

Some participants recognised the complexity of dual diagnosis of chronic pain and pOUD and described a regulatory system that had a dichotomous view of the patients, they were either pain patients or had pOUD who had to be treated with OAT. For the participants, this meant that pOUD diagnosis inexorably led to OAT, something that no patient wanted. To avoid this, they avoided the diagnosis of pOUD.

Administrative staff responding to demanding patients at reception added to a sense for some participants that they were powerless and this increased the chance that an opioid prescription would be written and decreased their ability to drive change.

*‘…they’re* (patients) *putting pressure on reception staff to make sure they’ve got the script. And so, I guess there’s that pressure to do what they wanted…and in the time they wanted it to be done. And I can feel that kind of balance of power on the doctor patient relationship. Switching more to them being in control, being more and more demanding and telling me what I was going to do, rather than me guiding them on optimal treatment and actually being able to help them make a change’* (GP18, female, metro, established GP).

Participants who currently prescribed OAT were less affected by the social norms of colleagues but were equally concerned about the patient’s desire not to be diagnosed.

#### Diagnosing/managing pOUD undertaken by others

GP colleagues who undertook OAT prescribing were seen as addiction colleagues not as mainstream GPs by non-OAT prescribing GPs.

*‘….she* (GP Colleague) *is the addiction specialist…’* (GP24, male, rural, established GP).

This suggests that treating pOUD was not normative for GPs. Participants had little experience of other GPs prescribing OAT. Those who did prescribe saw this as a professional responsibility rather than something they wanted to do.

*‘I’m not really interested in taking* (more of) *these* (OAT) *patients on …that’s just being honest.’* (GP10, male, rural, established GP).

*‘It’s not my forte in general practice and I must admit, this isn’t something I seek out.’* (GP22, female, metro, new fellow).

### Subjective behavioural controls diagnosing and managing pOUD

#### Internal behavioural controls

Many participants described lack of knowledge, skills and low confidence with diagnosis and management of pOUD in chronic pain patients. Many participants without addiction training did not know the criteria for the diagnosis of OUD.

*‘…it is something that I don’t know a lot about, I don’t see a lot of, I’m not doing it all day long…’* (GP 11, female, metro, established GP).

Younger participants suggested they would be happy to prescribe but did not have the knowledge and skills needed to do this.

*‘.it’s a knowledge and management thing rather than an I don’t want to do it. I just feel like I’m not sure how.’* (GP7, female, rural, registrar).

Many participants indicated that they felt unprepared to be involved in the management of pOUD. They suggested that patients with aberrant behaviours such as injecting and diverting medication needed addiction services and that they would not be able to manage these issues. For this group of GPs, patients exhibiting aberrant behaviour were negatively compared to chronic pain patients with dependence on pain medications.

*‘…if I’m suspecting substance abuse behaviours rather than dependence on the medication someone with chronic pain can have, then it changes things, I need to involve more of an addiction specialist, or addiction services rather than to continue prescribing myself…’* (GP 12, female, rural, established GP).

Referral to specialist services was considered by most participants. They suggested that they would tend to refer patients like the case study to pain specialists and would be reluctant to refer to drug and alcohol services.

*‘I haven’t done it* (referred to drug and alcohol) *for a long, long while, though…I probably haven’t had a lot of experience with it…’* (GP18, female, metro, established GP).

Concern and fear of perceived risks associated with prescribing OAT for pOUD in their chronic pain patients was a feature of many participants’ responses. They were concerned that prescribing OAT would lead to an influx of patients requesting this treatment and worried about being overwhelmed by this demand.

*‘I don’t necessarily want to open the floodgates to all of the people who might be interested or need my help in that zone because there’s so much of it around here, and I don’t think that I can treat or see them all and I’m scared that if I open up that door that it will be never ending.’* (GP2, female, regional, established GP).

#### External behavioural controls

Lack of time, money and support, were universal to the participants’ experience. They described how limited consultation time and poor remuneration stopped them from engaging in what they saw as difficult, time-consuming conversations. The lack of adequate remuneration suggested for them that GPs’ time and effort was not valued.

*‘…they’re long hard consults…not paid, as well as what you deserve to be remunerated for, you know how much effort you’re putting in and how much reward you’re getting financially is not great…at the end of the day …you want to feel valued…’* (GP10, male, rural, established GP).

Conversations with patients about their pain and opioid use were made easier with more consultation time for many participants.

*‘I think, framing things correctly, is more difficult when you don’t have time. Just having plenty of time available and having just that sense of calm. It just makes your difficult conversation much easier.’* (GP 9, male, outer metro, established GP).

Treatment affordability was described by many participants as an important barrier preventing many patients from accessing alternatives to opioids.

*‘…a lot of the alternative things that I can use though, are very restricted financially depending on your patient…’* (GP8, female, metro, established GP).

Participants working in private billing practices (government funded with additional patient co-payment) suggested a different experience compared to working in bulkbilling (wholly government funded) practices. These participants suggested their patients, who had higher levels of education, health literacy and better financial status, showed higher engagement with advised treatment options and greater ability to pay for more costly alternative treatments.

*‘a lot of our patients are very much about prevention and trying to get off medication…because we’re private clinic,…that changes the dynamic and… I would say probably (the) overwhelming majority of my patients have…. university degrees and they’re pretty well educated and…have high health literacy.’* (GP16, female, metro new fellow).

Low levels of specialist support were seen as a barrier to assisting patients with chronic pain and pOUD by most participants.

*‘I just don’t have necessarily have access to a chronic pain team or that kind of help…’* (GP7, female, rural registrar).

*‘…the couple of times I’ve tried to work with drug and alcohol. The doctor I’ve spoken to hasn’t been that helpful and so that’s made me more reluctant to talk with them, because it’s kind of feels like well wherever I turn my patients are getting knocked back. And so, it’s hard to access this specialist support for my patients.’* (GP18, female, metro, established GP).

One GP who expressed interest in providing OAT described how he was inundated by patients from the public addiction service and had to stop accepting referrals. This was compounded by the lack of promised support from the specialist service.

*‘I just got pummelled and eventually ended up saying, no. Sorry, I just don’t have the capacity to take on large numbers of patients, but also because the promise the system, the reality was always substantially less than the expectation, in terms of that support availability.’* (GP24, male, rural, established GP).

The role of specialist patient centred shared care and support was seen as a great advantage by many participants and one that could lead to better outcomes.

*‘I think it can be fantastic, obviously, to have a shared care arrangement where, especially with complex comorbidity, then the more people on the team and the more eyes on the situation, the better the outcome is for the patients, 100% having expert advice that’s accessible and patient centred is terrific.’* (GP11, female, metro, established GP).

Some suggested that they had good understanding of their patient within their context and knew what local services were available. They suggested the value of good professional relationships with their local pharmacists.

*‘I can ring my community pharmacist and go, Hey, what do you think about this person and their dosing? Do you think that there’s any issues or like, how do you think that they should go? …and I feel like I can trust them, I know them because they’re around the corner.’* (GP2, female, rural, established GP).

Many participants were worried that patients might experience stigma with other health professionals. This led to avoidance of using the term pOUD, with patients, in the medical record or letters to other services. They suggested that this may lead to inferior treatment by other health professionals.

*‘I don’t love labels…if I’m referring a patient to hospital,…I don’t want them to be discriminated against any way…’* (GP12, female, rural, established GP).

**Fig. 2:**
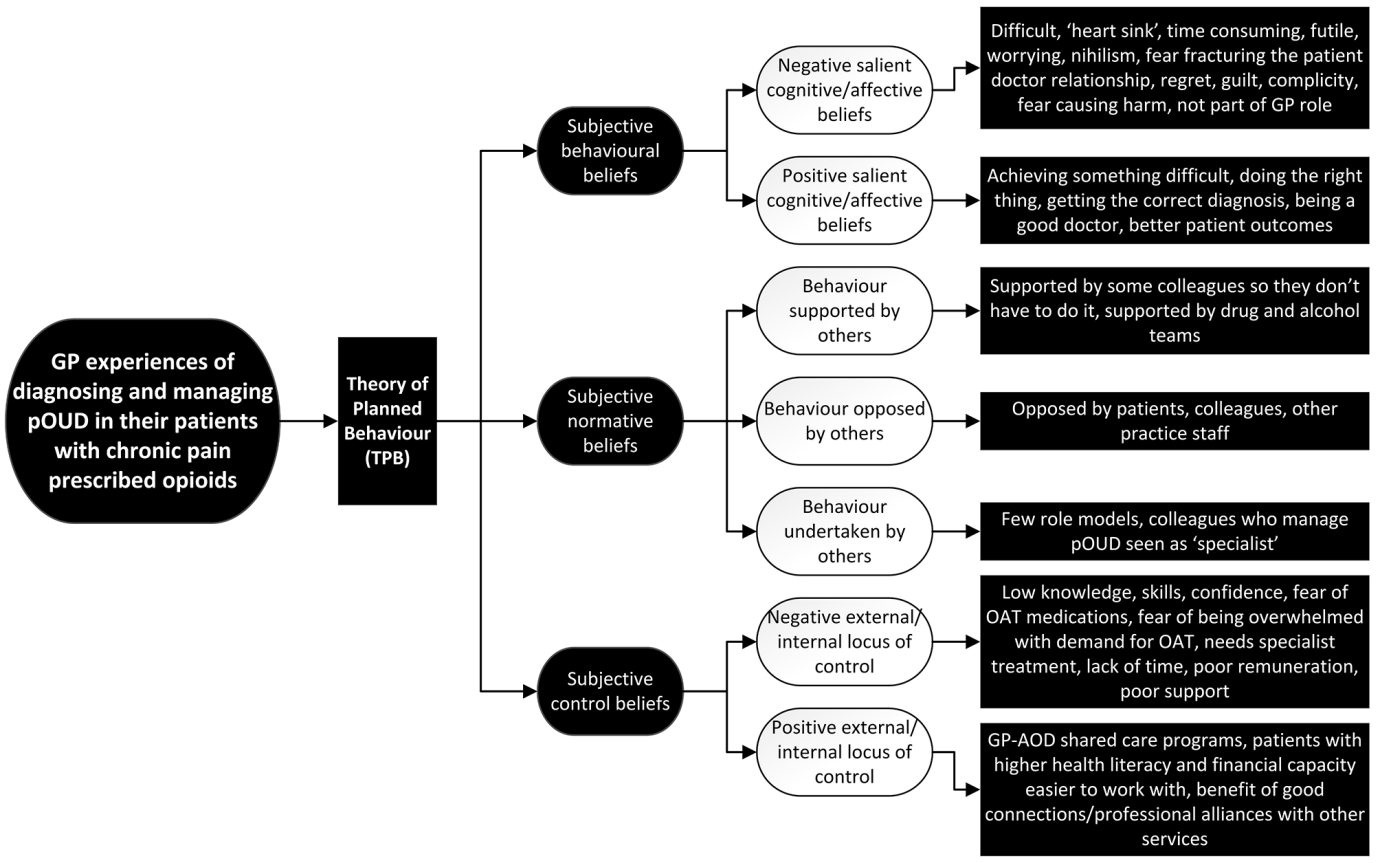
Theory of Planned Behaviour factors

The three TPB factors investigated in this study are summarised in Fig. 2 below.

## Discussion

This study, based on GP self-report, explored the subjective behavioural, normative and control beliefs that impact pOUD diagnosis and management in patients prescribed opioids for chronic pain. Overall, the beliefs expressed by the participants suggest there will be low intention and therefore low levels of actual diagnosis or management of pOUD if this develops in their chronic pain patients on opioids.

All participants responded to the scenario in the case study with a holistic generalist approach considering the impact of multiple biopsychosocial issues. They gave considered, thoughtful responses regarding their difficulties and their failings in their approach to working with patients prescribed opioids for chronic pain.

Participants expressed feelings of conflict and futility in the face of diagnosing and managing pOUD in their chronic pain patients. They described negative emotional experiences, discomfort and fear, and feelings of being complicit in causing harms to their patients. They suggested that diagnosing and managing pOUD was important, but this was outweighed by their past experiences of difficult conversations, difficult patients, fragile therapeutic alliances, a lack of sense of control and a sense of futility and powerlessness that they could positively influence their patients’ use of opioids. This led to avoidance of these conversations. Difficult conversations with patients experiencing chronic pain have been previously described in the literature [46], but to our knowledge, the difficulty of conversations around diagnosing and managing pOUD in patients prescribed opioids for chronic pain has not been studied.

Diagnosing and managing pOUD was not the norm for participants and impacted by lack of support from colleagues, practice staff and specialist services [32, 47]. The idea that staff did not want “these patients” in the practice belies the fact that patients with pOUD were already in the practice, just not yet diagnosed. Participants described positive support from some of their medical colleagues, but only because this enabled those colleagues to avoid diagnosing and managing pOUD themselves. Paramount was the lack of positive patient social norms. Participants believed that patients didn’t want to have these conversations, they didn’t want the diagnosis, or change in management. Participants believed that their patients saw themselves as pain sufferers, that they needed their opioids and did not want to consider management that would make them part of a stigmatised group of people with OUD.

Participants had few role models to provide them with a basis to undertake this behaviour. They expected to be left unsupported and unable to provide the level of care required for this chronic condition. Prescribing for pOUD was not seen as ‘normal’ work for many participants, but rather as specialist work, outside the responsibility of general practice. No one wanted this diagnosis, not the patient, not the participant and not the participant’s GP practice. The risk of ‘inundation’ that participants felt is compounded by a long standing lack of ODT prescribers [28] and the resulting unmet treatment need in Australia [48, 49].

Participants described low levels of knowledge, skill, and confidence as well as barriers including limited time, remuneration, little specialist support and difficult regulatory requirements. Internal and external behavioural controls to prescribing OAT; lack of skill, knowledge, confidence, time, remuneration and specialist support have been described in previous studies [31–34]. External controls also speak to systemic and structural issues, particularly time constraints that are integral to the ‘fee for service’ structure in Australian general practice [50].

Participants were highly aware of the risks associated with long term prescribed opioids [51, 52]. However, their knowledge of pOUD, the variety of treatments available and regulatory requirements was often incomplete. The task of re-considering treatment options required participants to re-orient their approach deliberately and consciously. This did not come easily. Putting limits and boundaries on patient opioid requests was conflictual. Negotiating a person-centred approach that did not give in to patient demand was perceived to be difficult. Participants considered the role of reducing opioid dose, changing treatment plan but avoided the diagnosis of pOUD as they felt they had to choose between continuing the status quo, or diagnosing pOUD, a diagnosis that they felt must inexorably lead to a difficult change in management and force them to move the patient to treatment with methadone or buprenorphine under the NSW OAT program [53], despite the fact that this is not mandatory. This decreased participants’ intent to diagnose and manage pOUD and dovetailed into the participants’ fear that they would be overwhelmed by demand.

Stigma is often cited as a reason GPs avoid treating addiction [54]. Experience of stigma and discrimination prevents people seeking or staying in care, leading to poorer treatment effectiveness and adverse patient outcomes [55]. Stigma and bias were important factors driving participant beliefs and intentions in this study. This was not simple and had two important aspects; participants’ lived experience of difficult conversations with patients at risk of pOUD and their concern about the risk of patient stigma and discrimination by colleagues and other health services. Past experience led to a tendency to believe that all future conversations would be conflictual, that all patients would be complex [56], when in fact there are a wide range of patient presentations and levels of stability [57]. Both past experience and concern about stigma from other services led to inertia and avoidance of the conversation and the diagnosis. Medicolegal concerns about the implications of diagnosis were important, however participants were also aware of the risk of not diagnosing pOUD, including medicolegal risk [58, 59]. On balance, the difficult emotional work, lack of social norms and adverse internal and external behavioural controls pushed them towards inaction, despite the risks.

### Strengths and limitations

This study examined the lived experience of GPs working in rural, regional and metro NSW. The participants spoke frankly about their difficulties. A qualitative method with a mid-range theory supported the study’s ability to do this as did the insider status of the GP interviewer.

Our participants included female GPs who tend to see more patients with complex and psychological issues [60] and younger GPs who may be more open to addressing addiction [61]. As a result, this group may be more open to the issue of pOUD in chronic pain and reluctance to diagnose and manage pOUD may be even stronger among other Australian GPs.

The study relied on participant’s self-report. Memory may have been selective, misattributed or exaggerated. Participants may have wished to appear more confident and comfortable than they really were. Social desirability bias may have led them to report what they felt they should do rather than what they actually do in practice. This may have been mitigated by the use of an experienced ‘insider’ interviewer; a GP who has experienced the issues and as a result was able to put participants at ease using a curious questioning style that encouraged frank discussion.

Australian State and Territory regulatory requirements limit access to OAT. In NSW, GPs can prescribe for up to 30 people without training and for 200 after training [62]. This is more liberal than other Australian jurisdictions, which have a varied range of prescriber restrictions. Given the complex barriers experienced by GPs in NSW, it is likely that less liberal rules in other jurisdictions will further negatively impact GPs’ willingness to prescribe OAT.TPB describes a framework for individual intention, and it is important to address systems issues that impact on behaviour, including societal stigma, fear and loathing of people with substance use disorders and lack of legitimacy for these as a chronic medical condition. Constraints including time, remuneration and regulatory requirements are both perceived and actual, they are structural and systemic. TPB cannot address this and is limited to individual intentions.

This research is limited to the experience of GPs and does not investigate the perspectives from other stakeholders such as patients, carers and policy makers.

## Conclusions

Our analysis suggests that there were major perceived barriers to diagnosing and managing pOUD in patients prescribed opioids for chronic pain by GPs in general practice in NSW, Australia. Negative attitudes, negative social norms and negative perceived behavioural controls lead to low intention to diagnose and manage pOUD, and therefore low chance that this will occur, a decision which is associated with potential significant harms. Without adequately addressing these barriers, we cannot hope to change this.

### Implications

Understanding GPs’ past negative experience and the influence of this on current behaviours is core to improving the diagnosis and management of pOUD in chronic pain patients prescribed opioids. It is essential to address not only the perceived behavioural controls such as time, remuneration and skills, but also to reduce the negative beliefs and strengthen appropriate social norms for GPs. These may be addressed by giving GPs opportunities to reflect on their patients with chronic pain through audit and education that includes building skills to manage difficult clinical interactions [63]. Repeated and early exposure to these complexities for doctors in training may assist. Ensuring people with lived experience of pOUD are involved in leading this training would be helpful as may building role models and champions [64] in primary health networks and GP colleges.

Additional support from specialist services to GPs (both in managing chronic pain and pOUD), training other team members in the practice on pOUD, including reception staff/practice managers, nurses, and allied health staff will ensure they have better understanding of the complexities of patients’ issues and skills to manage these. Providing a signal that this care is supported and valued through changes to funding mechanisms, i.e., creating specific Medicare item numbers for this treatment may also positively impact social norms.

Better understanding of the treatment options for people who develop pOUD for GPs with comorbidity (chronic pain and pOUD) treatment guidelines could improve knowledge and better nuanced regulatory approaches may support this.

There have been several policy changes in Australia including OUD prescribing guidelines, regulatory changes, and the introduction of real time prescription monitoring. It is unclear if these changes will be sufficient to change the frequency that pOUD is diagnosed and managed in general practice. Further investigation through the lens of TPB will help government, policy makers and service managers to assess the positive impact of these changes on this complex clinical presentation and GPs intention to diagnose and manage pOUD.

### Electronic supplementary material

Below is the link to the electronic supplementary material.

Supplementary Material 1

Supplementary Material 2

## Data Availability

The datasets generated and/or analysed during the current study are not publicly available due to the sensitive, confidential, and potentially re-identifiable nature of the semi structured interviews undertaken. Additionally, our ethics approvals does not allow disclosure of these data. More details are available from the corresponding author on reasonable request.

## Abbreviations

GP: General Practitioner
pOUD: Prescription Opioid Use Disorder
OAT: Opioid Agonist Treatment
AOD: Alcohol and Other Drugs
TPB: Theory of Planned Behaviour
