# Supplementary material for: Diagnosing and managing prescription opioid use disorder in patients prescribed opioids for chronic pain in Australian general practice settings: a qualitative study using the theory of Planned Behaviour
Source: BMC Prim Care. 2024 Jul 3;25:236. doi: 10.1186/s12875-024-02474-6 (PMC11223276; doi:10.1186/s12875-024-02474-6)
Supplement: Supplementary file 1 — Supplementary Material 1 [file 12875_2024_2474_MOESM1_ESM.docx]

**Supplemental File 1 - Interview Guide**

**Introduction:**Thank you/ your name/ purpose / confidentiality / duration / conduct of the interview / opportunity for questions / consent form signed/demographics

**Demographics -** Age, gender, years as GP, years in current practice, cultural background, rurality, no of patients with CP, no of patients prescribed opioids, post graduate training

**Question guide (note these questions will be asked if needed to probe the issues)**

**(For focus group members, explain the progression from the focus group to this phase)**

Introduce case study (part 1)– do you have patients similar to this? What do you think are the issues here? Is pOUD an issue? (introduce part 2 if needed) How might you diagnose this? What are the treatment options? How would you treat this?

**General beliefs regarding patients with pOUD**

What is your opinion of diagnosing and managing pOUD in general?

**Behavioural beliefs regarding diagnosing and managing pOUD**

What do you believe are the advantages of diagnosing and managing pOUD?

What do you believe are the disadvantages of diagnosing and managing pOUD?

**Normative beliefs regarding prescribing**

Are there any individuals or groups who would approve of your decision to diagnose and manage pOUD?

Are there any individuals or groups who would disapprove of your decision to diagnose and manage pOUD?

Are there any other individuals or groups who put pressure on you when you are deciding whether to diagnose and manage pOUD?

**Control beliefs regarding prescribing**

What factors or circumstances would enable you to diagnose and manage pOUD?

What factors or circumstances would make it difﬁcult or impossible for you to diagnose and manage pOUD?

Are there any other issues that come to mind when you think about diagnosing and managing pOUD

Awareness of current policies and strategies to support GPs opioid prescribing

**Closing**

Is there anything else you would like to tell me? Give thanks/intention to share key insights and a copy of the final paper if interested.
