# Supplementary material for: Diagnosing and managing prescription opioid use disorder in patients prescribed opioids for chronic pain in Australian general practice settings: a qualitative study using the theory of Planned Behaviour
Source: BMC Prim Care. 2024 Jul 3;25:236. doi: 10.1186/s12875-024-02474-6 (PMC11223276; doi:10.1186/s12875-024-02474-6)
Supplement: Supplementary file 2 — Supplementary Material 2 [file 12875_2024_2474_MOESM2_ESM.docx]

**Supplemental File 2 – CORE-Q**

CORE-Q Consolidated Criteria for Reporting Qualitative Research

File name: Supplemental file 2

File format: Word

Title of data: CORE-Q Consolidated Criteria for Reporting Qualitative Research

Description of data: Table reporting on the characteristics of this qualitative research using standardised criteria.

| **No** | **Item** | | **Description** |
| --- | --- | --- | --- |
| **Domain 1: Research Team and Reflexivity** | | | |
|  | **Personal Characteristics** | | |
| 1 | Interviewer / facilitator | The principal researcher and interviewer (HW) is an experienced GP and has extensive experience of working as a GP in the Medicare billing community practice space and firsthand experience of the challenges of assisting people with chronic pain and opioid use in Australian general practice. She was known to the participants as a colleague and fellow GP and lead in the alcohol and other drug primary care field.  Other researchers are HW’s PhD supervisor, (MFH) co supervisor (BHR) and associate supervisor (NL). | |
| 2 | Credentials | HW is a PhD candidate with extensive experience working as a GP and in drug and alcohol settings.  MFH is a professor of General Practice with extensive clinical and research experience in general practice.  BHR is an associate professor with extensive experience in primary care and health service research  NL is an associate professor with extensive clinical and research experience in addiction. | |
| 3 | Occupation | 3 researchers are clinicians, and all are researchers. There is experience in this team with both quantitative and qualitative research. | |
| 4 | Gender | Female, male, male, male | |
| 5 | Experience and Training | Extensive clinical experience and credibility for all clinical members of the research team amongst participants | |
|  | **Relationship with participants** | | |
| 6 | Relationship established | HW is known to participants | |
| 7 | Participant knowledge of the interviewer | Known to be a fellow GP with similar work experience and additional experience and expertise in AOD issues | |
| 8 | Interviewer characteristics | Experienced clinical interviewer, educator and clinician | |
| **Domain 2: Study Design** | | | |
|  | **Theoretical Framework** | | |
| 9 | Methodological orientation and theory | Qualitative, framed through the Theory of Planned Behaviour | |
|  | **Participant Selection** | | |
| 10 | Sampling | Purposive, i.e. GPs working in primary care settings in NSW | |
| 11 | Method of approach | Via EOI email through Central and Eastern Sydney Primary Health Network (CESPHN), 2400 members.  Via Facebook post on GPDU (GPs down under). GPDU is a national private Facebook page with more than 7000 active members nationally. The moderators allowed recruitment for this ethics approved study.  Interested GPs replied via email or via direct message.  Participants encouraged other GPs to also take part. (snowballing) | |
| 12 | Sample size | CESPHN 2400 members  GPDU 7000 members | |
| 13 | Non participation | To participate had to be GP working in community general practice in NSW. Two interstate GPs applied and were excluded. The study was limited to NSW due to the variation in state based MATOD prescribing legislation. | |
|  | **Setting** | | |
| 14 | Setting of data collection | All GPs working in general practice in NSW were invited to take part in semi structured interviews via an online video conferencing platform (Zoom). Interviews ran for 45-60 minutes.  The invite indicated that the researchers were interested in a wide range of views and experience and this helped to encourage a mix of gender, age, GP experience (from early career to extended mature career GPs) and GPs with varied interest (from specific interest to little interest) in AOD care.  We were keen to understand the experience of a diverse group of GPs working in metropolitan, rural and regional GP settings. | |
| 15 | Presence of non-participants | No non participants were present during the interviews | |
| 16 | Description of sample | 24 participants undertook 24 individual semi structured interviews | |
|  | **Data Collection** | | |
| 17 | Interview guide | A case study (see supplemental file 1) was used with the interview guide – see below  **Introduction:**Thank you/ your name/ purpose / confidentiality / duration / conduct of the interview / opportunity for questions / consent form signed/demographics  **Demographics -** Age, gender, years as GP, years in current practice, cultural background, rurality, no of patients with CP, no of patients prescribed opioids, post graduate training  **Question guide (note these questions will be asked if needed to probe the issues)**  **(For focus group members, explain the progression from the focus group to this phase)**  Introduce case study (part 1)– do you have patients similar to this? What do you think are the issues here? Is pOUD an issue? (introduce part 2 if needed) How might you diagnose this? What are the treatment options? How would you treat this?  **General beliefs regarding patients with pOUD**  What is your opinion of diagnosing and managing pOUD in general?  **Behavioural beliefs regarding diagnosing and managing pOUD**  What do you believe are the advantages of diagnosing and managing pOUD?  What do you believe are the disadvantages of diagnosing and managing pOUD?  **Normative beliefs regarding prescribing**  Are there any individuals or groups who would approve of your decision to diagnose and manage pOUD?  Are there any individuals or groups who would disapprove of your decision to diagnose and manage pOUD?  Are there any other individuals or groups who put pressure on you when you are deciding whether to diagnose and manage pOUD?  **Control beliefs regarding prescribing**  What factors or circumstances would enable you to diagnose and manage pOUD?  What factors or circumstances would make it difﬁcult or impossible for you to diagnose and manage pOUD?  Are there any other issues that come to mind when you think about diagnosing and managing pOUD?  Awareness of current policies and strategies to support GPs opioid prescribing  **Closing**  Is there anything else you would like to tell me? Give thanks/intention to share key insights and a copy of the final paper if interested. | |
| 18 | Repeat interviews | Planned | |
| 19 | Audio/visual recording | audio | |
| 20 | Field notes | yes | |
| 21 | Duration | 45-60 minutes per interview | |
| 22 | Data saturation | No new themes by end of 20 interviews. 4 additional interviews were run to ensure this was the case. | |
| 23 | Transcripts returned | Transcripts were not shared with participants. Key insights were shared and final publications. | |
| **Domain 3: Analysis and Findings** | | | |
|  | **Data Analysis** | | |
| 24 | Number of data coders | 1 major and 3 reviewers | |
| 25 | Description of the coding tree | Deductive coding of by 3 facets of TPB, led to 11 overarching themes. See figure 1 for a visual representation of this. Note this figure combines 8 themes into 4 covering negative and positive aspects of the themes. | |
| 26 | Derivation of themes | Deductive based on theoretical framework of TPB | |
| 27 | Software | NVivo 12 | |
| 28 | Participant checking | No, however member checking carried out with BHR, MFH and NL | |
|  | **Reporting** | | |
| 29 | Quotations presented | yes | |
| 30 | Data and findings consistent | yes | |
| 31 | Clarity of major themes | yes | |
| 32 | Clarity of minor themes | yes | |
